# The role of blood pressure variability indicators combined with cerebral blood flow parameters in predicting intraventricular hemorrhage in very low birth weight preterm infants

**DOI:** 10.3389/fped.2023.1241809

**Published:** 2023-10-09

**Authors:** Lijun Jiang, Qian Yu, Fudong Wang, Mingfu Wu, Feng Liu, Mingfeng Fu, Junyan Gao, Xing Feng, Longfeng Zhang, Zhenxing Xu

**Affiliations:** ^1^ Department of Neonatology, Affiliated Hospital of Yangzhou University, Yangzhou, China; ^2^ Department of Neonatology, Affiliated Children's Hospital of Soochow University, Suzhou, China; ^3^ Department of Clinical Laboratory, Affiliated Hospital of Jiangsu University, Zhenjiang, China

**Keywords:** preterm infant, intraventricular hemorrhage, blood pressure, blood pressure variability, ACA resistance index, transcranial Doppler

## Abstract

**Background:**

Hemodynamic instability is the main factor responsible for the development of intraventricular hemorrhage (IVH) in premature newborns. Herein, we evaluated the predictive ability of blood pressure variability (BPV) and anterior cerebral artery (ACA) blood flow parameters in IVH in premature infants with gestational age (GA) ≤32 weeks and birth weight (BW) ≤ 1,500 g.

**Methods:**

Preterm infants with GA ≤32 weeks and BW ≤ 1,500 g admitted to the neonatal intensive care unit (NICU) of the hospital affiliated to Yangzhou University from January 2020 to January 2023 were selected as the research subjects. All preterm infants were admitted within 1 h after birth, and systolic blood pressure (SBP), diastolic blood pressure (DBP), and mean arterial blood pressure (MABP) were monitored at 1-h intervals. The difference between maximum and minimum values (max-min), standard deviation (SD), coefficient of variation (CV), and successive variation (SV) were used as BPV indicators. On the 1st, 3rd, and 7th day after birth, transcranial ultrasound examination was performed to screen for the occurrence of IVH. On the 24 ± 1 h after birth, systolic velocity (Vs), diastolic velocity (Vd), and resistance index (RI) of the ACA were measured simultaneously. Preterm infants were divided into the IVH group and non-IVH group based on the results of transcranial ultrasound examination, and the correlation between BPV indicators, ACA blood flow parameters, and development of IVH was analyzed.

**Results:**

A total of 92 premature infants were enrolled, including 49 in the IVH group and 43 in the non-IVH group. There was no statistically significant difference in baseline characteristics such as BW, GA, sex, and perinatal medical history between the two groups of preterm infants (*P* > 0.05). The SBP SD (OR: 1.480, 95%CI: 1.020–2.147) and ACA-RI (OR: 3.027, 95%CI: 2.769–3.591) were independent risk factors for IVH in premature newborns. The sensitivity and specificity of combined detection of SBP SD and ACA-RI in predicting IVH were 61.2% and 79.1%, respectively.

**Conclusion:**

High BPV and ACA-RI are related to IVH in premature infants with GA ≤32 w and BW ≤1,500 g. Combined detection of SBP SD and ACA-RI has a certain predictive effect on early identification of IVH.

## 1. Introduction

Intraventricular hemorrhage (IVH) is a common form of brain injury in premature infants, leading frequently to adverse neurodevelopmental outcomes or death (1–3). In developing countries, the survival rate of very low-birth weight (VLBW) infants has been continuously improving in recent years, while the incidence of IVH has not decreased (4). Studies have shown that 20%–25% of VLBW preterm infants develop IVH, while the incidence of IVH in extremely low-birth weight (ELBW) infants is as high as 45% (1, 4). IVH can not only cause acute death in preterm infants but also leave varying degrees of neurological sequelae (5, 6).

Currently, it is believed that IVH is the result of multiple harmful factors acting together on the basis of immaturity, among which unstable cerebral blood flow is one of the important factors (2). The immaturity of cerebral vascular autoregulation function in preterm infants, as well as the passive cerebral blood flow caused by high or low peripheral blood pressure (BP) is related to the onset of IVH in premature infants (7, 8). Therefore, maintaining cerebral hemodynamic stability is particularly important for the clinical management of IVH. Currently, there is no consensus on normal BP in preterm infants (9, 10), and there are still controversies in evaluating cerebral blood flow stability using the optimal range of blood pressure alone (11–14). Blood pressure variability (BPV) refers to the degree to which BP fluctuates over a period of time. BPV can better reflect the hemodynamic state of the body than BP (15). In recent years, research has found that BPV is closely related to brain damage caused by fluctuations in adult cerebral blood flow (16). Therefore, we speculate that BPV analysis and monitoring of cerebral blood flow parameters can provide new references for early prediction of IVH in premature infants.

## 2. Materials and methods

### 2.1. Patient selection

Premature infants with gestational age (GA) ≤32 weeks and birth weight (BW) ≤1,500 g enrolled at the NICU of the hospital affiliated to Yangzhou University from January 2020 to January 2023 were selected as the research subjects. All infants were admitted to the NICU within 1 h after birth, and were included in either the IVH group or the non-IVH group according to the results of transcranial ultrasound imaging. Infants were excluded if they suffered from congenital brain developmental abnormalities and central nervous system infections. This study meets the requirements of medical ethics and has been reviewed by the hospital's medical ethics committee (approval number: 2022-YKL3-06-005).

### 2.2. General data

Demographic and clinical information were collected for all enrolled infants, including GA, BW, sex, perinatal medical history, mode of ventilatory support, antenatal steroids use, pulmonary surfactant (PS) administration, patent ductus arteriosus (PDA), and incidence of early-onset sepsis, and so on.

### 2.3. Imaging

All the infants underwent transcranial ultrasound on the 1st, 3rd, and 7th day after birth, and the Papile's method was used to grade the diagnosis of IVH (17). Transcranial ultrasound was done using the 3–11 MHz, C11-3S probe, GE Vivid iq ultrasonic diagnostic apparatus, and examinations were carried out and classified by two senior neonatologists with at least 5-year experience in neonatal transcranial ultrasound.

On the 24 ± 1 h after birth, the transcranial Doppler simultaneously measured the peak systolic velocity (Vs) and maximum end-diastolic blood flow velocity (Vd) of ACA by placing the transducer in the mid-sagittal plane via the anterior fontanelle and using the pulsed wave doppler. The ACA resistance index (ACA-RI) was calculated using the following formula: ACA-RI = Vs - Vd/Vs.

### 2.4. Blood pressure data collection

All included infants underwent electrocardiogram monitoring by the GE Dash 3,000 multi-parameter monitor system immediately after admission, and 24-h ambulatory blood pressure monitoring was conducted every 1 h in the first three days of life. The monitoring indicators included systolic blood pressure (SBP), diastolic blood pressure (DBP), and mean arterial blood pressure (MABP). Blood pressure was measured using an oscillometric device with a cuff, and the width of the cuff to the arm circumference ratio closest to 0.50. We used the right upper arm to measure the blood pressure and the measurements were done during sleep or in a quiet awake state. In case of the newborn was not calm or crying during measurement, the remeasurements were done in a settled state. The value of BP was the average of at least 3 readings.

The difference between the maximum and minimum values of blood pressure (max-min), standard deviation (SD), coefficient of variation (CV), and successive variation (SV) were used as indicators of blood pressure variability (18, 19).

### 2.5. Statistical analysis

All statistical analyses were performed using SPSS software (version 26.0, IBM Corporation, Armonk, NY, USA). For continuous variables, Student's *t*-test was used for parametric testing and Mann–Whitney *U*-test was used for nonparametric testing. For categorical variables, the chi-square test was used. Univariate analyses were performed to identify possible risk factors that might be associated with IVH individually. Multicollinearity was tested among all factors identified to be possibly associated with IVH (*p* < 0.05). In the multivariate analysis, variables with collinearity (SBP CV and DBP CV) were excluded, and the remaining variables were further entered into the logistic regression model to determine independent predictors of IVH. The performance of factors identified as significantly related to IVH development from the regression analysis were assessed by receiver operator characteristic curve analysis and estimation of the corresponding AUC.

## 3. Results

### 3.1. Demographic and clinical data

**Figure 1:**
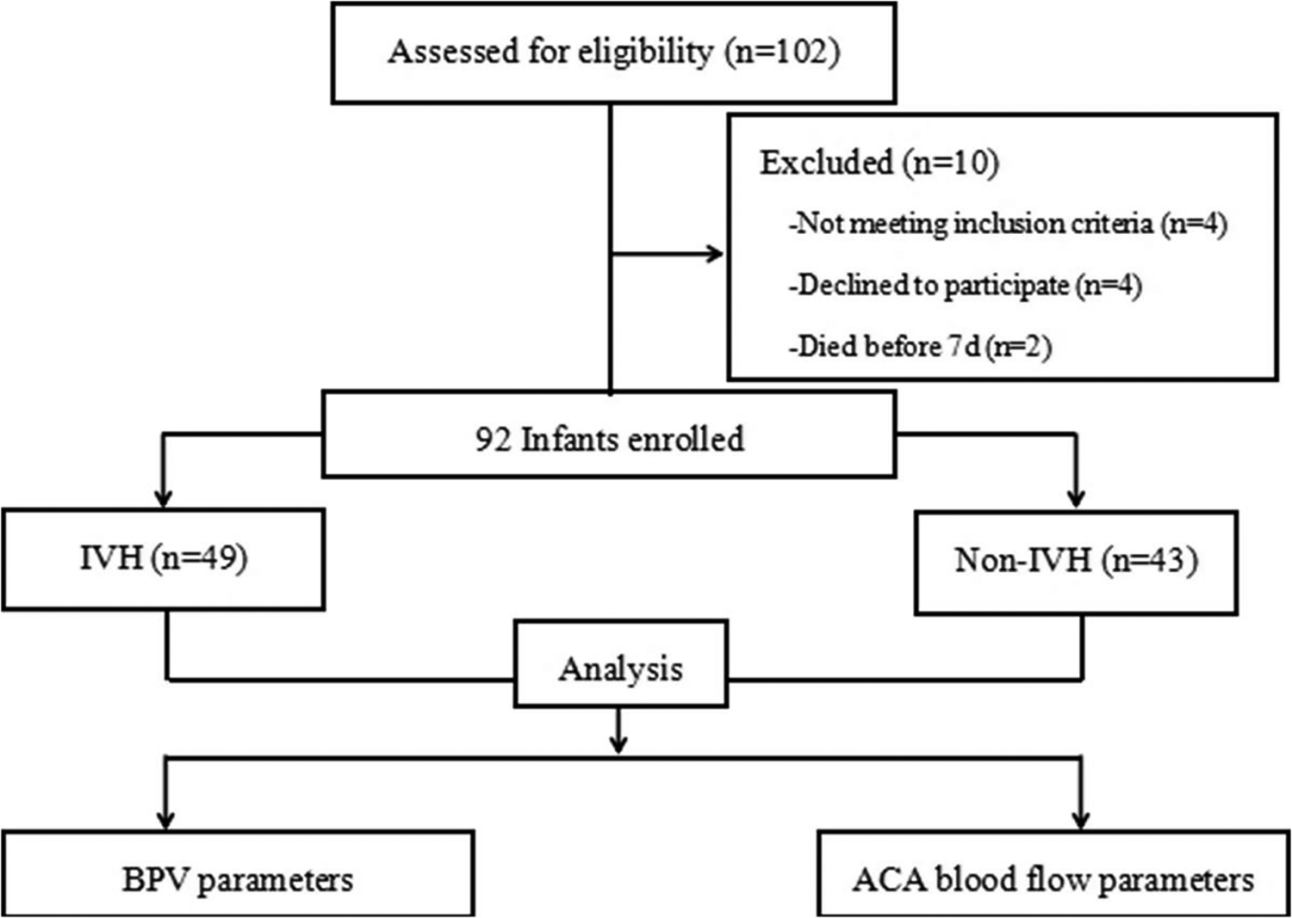
Study flow chart.

A total of 102 infants were admitted during the research window, 92 of whom met the inclusion criteria and completed the study (Figure 1). For the enrolled infants, the mean GA was 28.95 ± 2.33 weeks, and mean BW was 1,202.49 ± 251.51 g. A total of 41 infants were diagnosed with mild IVH, 8 infants with severe IVH, and 43 infants had no IVH in first seven days after birth (Tables 1, 2).

**Table 1:** Comparison of baseline data of preterm infants with and without IVH.

Characteristics	Non-IVH (*n* = 43)	IVH (*n* = 49)	*t/χ^2^*	*P*-value
Gestational age (weeks)	29.4 ± 2.1	28.6 ± 2.4	1.663	0.100
Birth weight (g)	1,252.1 ± 230.7	1,158.9 ± 263.1	1.794	0.076
Intrauterine growth retardation, *n* (%)	6 (14.0)	3 (6.1)	0.828	0.363
Gender (male), *n* (%)	22 (51.2)	26 (53.1)	0.033	0.856
Vaginal delivery, *n* (%)	20 (46.5)	27 (55.1)	0.676	0.411
Multiple birth, *n* (%)	3 (7.0)	6 (12.2)	0.247	0.619
Gestational diabetes mellitus	5 (11.6)	3 (6.1)	0.318	0.573
Gestational hypertension	8 (18.6)	5 (10.2)	0.730	0.393
Preeclampsia	3 (7.0)	2 (4.1)	0.023	0.881
Apgar at 5′≤7	5 (11.6)	13 (26.5)	3.232	0.072
Endotracheal tube in resuscitation	2 (4.7)	8 (16.3)	2.130	0.144
Invasive ventilation, *n* (%)	3 (7.0)	9 (18.4)	2.620	0.106
Antenatal corticosteroids, *n* (%)	29 (67.4)	26 (53.1)	1.970	0.160
Premature rupture of membrane, *n* (%)	12 (28.0)	16 (32.7)	0.244	0.622
Patent ductus arteriosus, *n* (%)	21 (48.8)	33 (67.3)	3.236	0.072
Surfactant therapy, *n* (%)	18 (41.9)	26 (53.1)	1.151	0.283
Early-onset sepsis, *n* (%)	6 (14.0)	11 (22.4)	1.097	0.295
Periventricular leukomalacia, *n* (%)	4 (9.3)	14 (28.6)	5.403	0.020
Inotropic drugs	3 (7.0)	10 (20.4)	3.405	0.065
NCIS	95.5 ± 9.4	93.8 ± 7.1	0.67	0.520
Mortality, *n* (%)	2 (4.7)	5 (10.2)	0.370	0.543

IVH, intraventricular hemorrhage. MAP, mean airway pressure. NCIS, neonatal critical illness score.

**Table 2:** Number of premature infants for the day of IVH diagnosis.

IVH Grade	Day of IVH diagnosis					Total
	D1	D3		D7		
Grade I–II	25 (−5)	18 (−2)		5		41
		Onset	Progression	Onset	Progression	
Grade III–IV	0	1	5	0	2	8
Total						49

−5, −2 is the number of premature infants with IVH progression from Grade I–II.

### 3.2. Blood pressure variability and IVH

The DBP and MABP of infants in the IVH groups were significantly lower than those in the non-IVH group (26.67 ± 5.38 mmHg vs. 28.86 ± 4.71, *p* = 0.042; 34.71 ± 5.75 vs. 37.09 ± 4.52 mmHg, *p* = 0.032), respectively, and the max-min, SD, and CV of SBP were significantly higher (24.86 ± 6.92 vs. 21.67 ± 7.32, *p* = 0.035); (6.22 ± 1.68 vs. 5.42 ± 1.59, *p* = 0.022); (11.39 ± 3.59% vs. 9.98 ± 3.05, *p* = 0.045), respectively. The max-min, SD, and CV of DBP were also significantly increased in the IVH group compared to the non-IVH group (26.43 ± 5.46 vs. 21.74 ± 7.76, *p* = 0.001); (6.70 ± 1.31 vs. 5.61 ± 1.76, *p* = 0.001); (26.06 ± 7.15% vs. 20.22 ± 8.28, *p* < 0.001), respectively (Table 3).

**Table 3:** Comparison of blood pressure variability parameters of preterm infants with and without IVH.

	Non-IVH (*n* = 43)	IVH (*n* = 49)	*t*	*P*-value
SBP (mmHg)	56.35 ± 4.68	55.78 ± 6.01	0.505	0.615
DBP (mmHg)	28.86 ± 4.71	26.67 ± 5.38	2.061	0.042
MABP (mmHg)	37.09 ± 4.52	34.71 ± 5.75	2.185	0.032
SBP max-min	21.67 ± 7.32	24.86 ± 6.92	2.142	0.035
SD	5.42 ± 1.59	6.22 ± 1.68	2.335	0.022
CV (%)	9.98 ± 3.05	11.39 ± 3.59	2.015	0.045
SV	8.17 ± 1.25	8.34 ± 1.37	0.609	0.544
DBP max-min	21.74 ± 7.76	26.43 ± 5.46	3.306	0.001
SD	5.61 ± 1.76	6.70 ± 1.31	3.310	0.001
CV (%)	20.22 ± 8.28	26.06 ± 7.15	3.626	<0.001
SV	7.49 ± 1.17	7.71 ± 1.47	0.810	0.420

SBP, systolic blood pressure; DBP, diastolic blood pressure; MABP, mean arterial blood pressure; max, maximum; min, minimum; SD, standard deviation; CV, coefficient of variation; SV, successive variation; IVH, intraventricular hemorrhage.

### 3.3. ACA blood flow parameters and IVH

Concerning imaging-based hemodynamic parameters, the ACA-Vd velocity was significantly lower (4.48 ± 2.58 vs. 5.76 ± 2.22 cm/s, *p* = 0.013) and the ACA-RI was significantly higher (0.79 ± 0.12 vs. 0.70 ± 0.11, *p* < 0.001) in the IVH group than the non-IVH group (Table 4).

**Table 4:** Blood flow parameters of ACA in preterm infants with and without IVH.

	Non-IVH (*n* = 43)	IVH (*n* = 49)	*t*	*P*-value
ACA Vs (cm/s)	20.36 ± 4.10	20.17 ± 3.68	0.226	0.822
ACA Vd (cm/s)	5.76 ± 2.22	4.48 ± 2.58	2.538	0.013
ACA RI	0.70 ± 0.11	0.79 ± 0.12	3.624	<0.001

ACA, anterior cerebral artery; Vs, systolic velocity; Vd, diastolic velocity; RI, resistance index; IVH, intraventricular hemorrhage.

### 3.4. Relationship between BPV and ACA-RI

**Figure 2:**
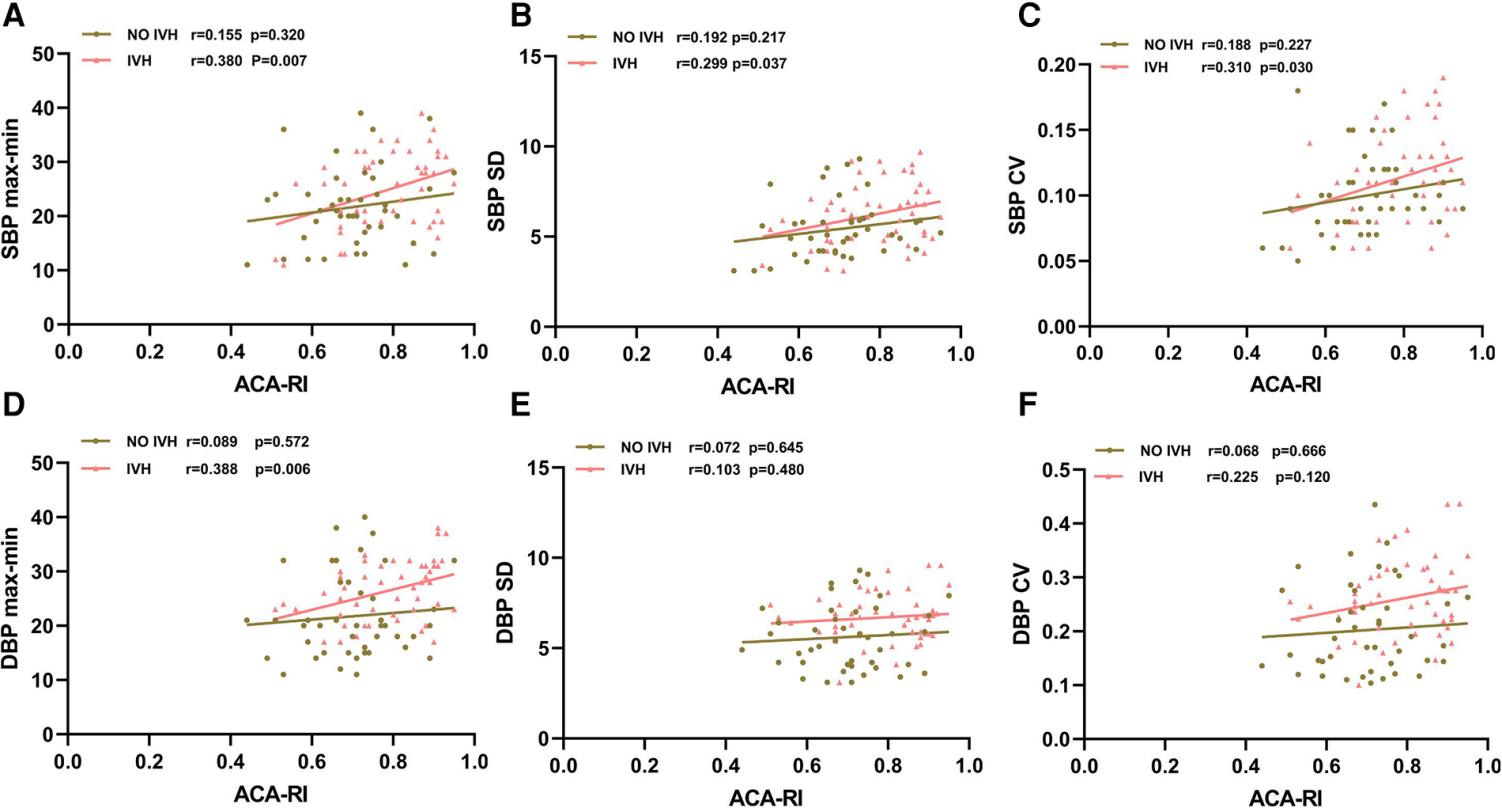
Relationship between SBPmax-min (**A**), SBP SD (**B**), SBP CV (**C**), DBPmax-min (**D**), DBP SD (**E**), DBP CV (**F**) and ACA-RI. SBP, systolic blood pressure; DBP, diastolic blood pressure; max, maximum; min, minimum; SD, standard deviation; CV, coefficient of variation; ACA, anterior cerebral artery; RI, resistance index; IVH, intraventricular hemorrhage. The red line with triangle markers represents the IVH group, and the brown line with round markers represents the non-IVH group.

The SBPmax-min, SBP SD, SBP CV, and DBPmax-min of infants in the IVH group were positively correlated with ACA-RI (*p* < 0.05), while the BPV indicators of SBP and DBP in the non IVH group were not correlated with ACA-RI (*p* > 0.05) (Figure 2).

### 3.5. Logistic regression analysis of risk factors for IVH

**Figure 3:**
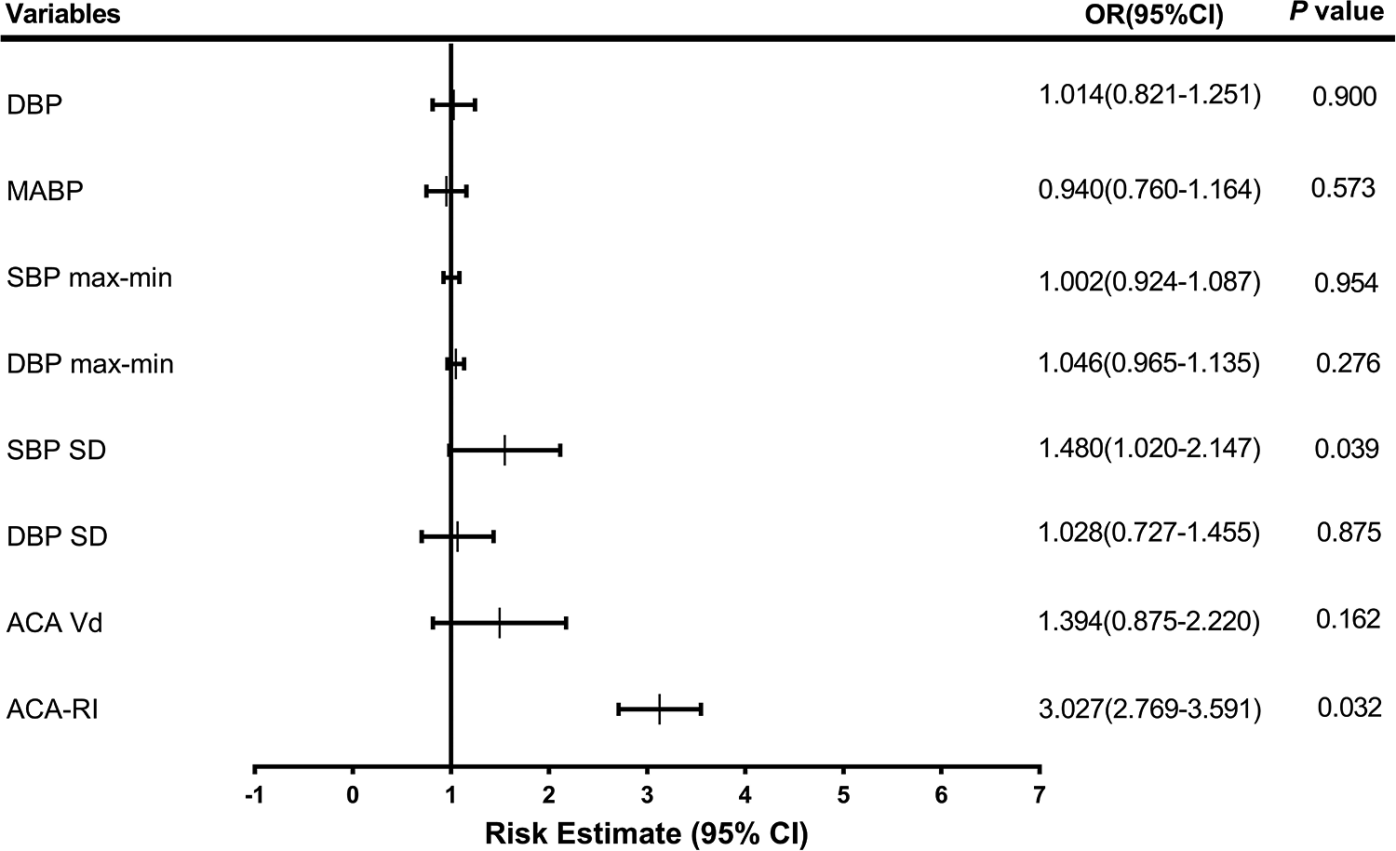
Odds ratios (OR) for neonates with intraventricular hemorrhage. DBP, diastolic blood pressure; MABP, mean arterial blood pressure; SBP, systolic blood pressure; max, maximum; min, minimum; SD, standard deviation; ACA, anterior cerebral artery; Vd, diastolic velocity; RI, resistance index.

In multiple regression analysis, SBP SD and ACA-RI were the most significant independent factors associated with IVH, with an OR (95% CI) of 1.480 (1.020–2.147) and 3.027 (2.769–3.591), respectively (Figure 3).

### 3.6. ROC curve analysis of risk factors for IVH

**Figure 4:**
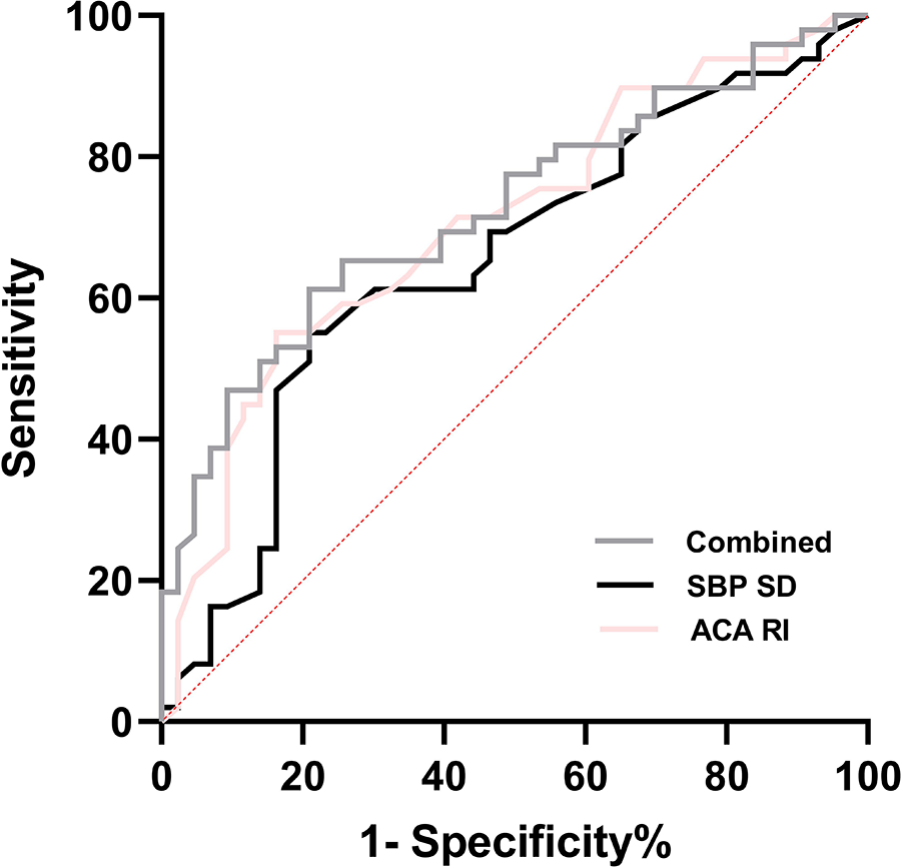
ROC curve of BPV parameters and ACA-RI for the prediction of IVH in premature infants. Combined = SBP SD + ACA RI; SBP, systolic blood pressure; SD, standard deviation; ACA, anterior cerebral artery; RI, resistance index.

The ROC curve analysis showed that in predicting IVH, the optimal cut-off value for ACA-RI was 0.79, with a sensitivity and specificity of 55.1% and 83.7%, respectively. The optimal cut-off value for SBP SD was 6.05, with a sensitivity and specificity of 55.1% and 79.1%, respectively. The combined detection of SBP SD and ACA-RI yielded the highest AUC of 0.725, with a sensitivity and specificity of 61.2% and 79.1% for predicting IVH in preterm infants (Figure 4).

### 3.7. Comparison of SBP SD and ACA RI in subgroups

Infants in the IVH group were further subdivided into mild IVH (grades I and II) and severe IVH (grades III and IV). The study found that compared with the non IVH group and the mild IVH group, the severe IVH group had significantly higher SBP SD, while ACA RI was significantly higher in the mild and severe IVH groups than in the non IVH group (Table 5).

**Table 5:** Comparison of SBP SD and ACA RI in subgroups.

	Non-IVH (*n* = 43)	IVH (*n* = 49)		*F*	*P*
		Grade I–II (*n* = 41)	Grade III–IV (*n* = 8)		
SBP SD	5.42 ± 1.59	5.84 ± 1.60	8.15 ± 1.20^a^^,^^b^	10.845	<0.001
ACA RI	0.70 ± 0.11	0.77 ± 0.12^a^	0.83 ± 0.06^a^	7.666	0.001

SBP, systolic blood pressure; SD, standard deviation; ACA, anterior cerebral artery; RI, resistance index.

^a^ *P* < 0.05, vs. Non-IVH group.

^b^ *P* < 0.05, vs. Grade I–II IVH group.

## 4. Discussion

In this study, we demonstrated that high BPV and ACA-RI are related to the development of IVH in premature infants with GA ≤32 w and BW ≤1,500 g, and that combined detection of SBP SD and ACA-RI has a certain predictive effect on early identification of IVH.

IVH is a common cause of neonatal brain injury, often associated with early death and long-term neurological developmental disorders in preterm infants (20, 21). The pathogenesis of IVH is complex and may be the result of a combination of multiple factors, among which the dysfunction of cerebral blood flow (CBF) caused by immature or dysregulated cerebral vascular autoregulation (CAR) function is an important factor (22). Preterm newborns are exposed to various hemodynamic instability factors after birth, which may further affect CBF perfusion, leading to a “fluctuating” CBF that could cause further risk of high perfusion and low perfusion brain injury (23).

The relative stability of CBF is particularly important for preterm infants. Although premature infants have established an autonomous regulatory mechanism for CBF, they are more susceptible to regulatory dysfunction caused by other factors (24). The use of hypotension or hypertension alone to indirectly reflect abnormal CBF dynamics in preterm infants may not be accurate (25). However, detection of cerebrovascular function based on imaging and continuous monitoring of BP fluctuations may be more accurate in predicting CBF impairment and IVH. Therefore, this study explored the capability of BP changes monitoring and cerebral hemodynamic measures in predicting the development of IVH in premature infants with GA ≤32 weeks and BW ≤1,500 g.

Our study found that the average values of MABP and DBP in the IVH group were significantly lower than the non-IVH group, consistent with previous research findings that IVH is associated with hypotension in preterm infants (26, 27). According to Bada et al.'s study (28), the MABP of newborns with grade II–IV IVH was significantly reduced compared to those without or with grade I IVH. However, there is currently no consensus on normal BP in premature infants, and the ideal BP range in this population is still controversial (9, 10). There are also studies that have shown that traditional definitions of hypotension (MABP < GA or MABP < 30 mmHg) are not associated with severe IVH (7). Therefore, using hypotension alone to indirectly predict IVH in preterm infants caused by low CBF perfusion may not be comprehensive (7). Hypertension and abnormal blood pressure fluctuations also affect CBF perfusion in premature infants, leading to the occurrence of IVH (29–31).

BPV can reduce the influence of pathological or physiological factors, exactly reflect the fluctuation of blood pressure, and evaluate changes in cerebral perfusion pressure (32). At present, BPV is widely used in the research of adult cardiovascular diseases, but its research on IVH in newborns, especially preterm infants, has not yet been reported. In our study on the relationship between BPV and IVH, we found that the IVH group had a higher BPV than the non-IVH group (except for SV) (*p* < 0.05), indicating that the greater the fluctuation of BP, the more likely a premature infant was to develop IVH. This indicates a certain correlation between high BPV and IVH development. We speculate that the systemic circulation fluctuation reflected by abnormal decrease or increase of blood pressure exceeds the physiological regulation range of CAR in preterm infants, and eventually leads to CBF disorder. Previous studies have confirmed that in preterm infants with IVH, there are blood pressure fluctuations exceeding the optimal regulatory range (7, 23), significant deviations below the optimal MABP are associated with death, and significant deviations above the optimal MABP are related to severe IVH (33).

In premature infants, alterations in cerebrovascular hemodynamics and fluctuations in CBF velocity increase the risk of intracranial hemorrhage (34, 35). At present, it is difficult to accurately evaluate CBF clinically. Some studies have used the superior vena cava flow (SVCF) to indirectly reflect CBF and have concluded that the fluctuation of SVCF is a critical risk factor for IVH (18, 24), and the stability of SVCF can avoid IVH (19, 36). Some research studies have also discussed the use of other parameters to reflect cerebral blood perfusion, such as cerebral fractional oxygen extraction (CFOE), peripheral tissue perfusion, and some biomarkers (20, 37). The above research methods are relatively complex, and there are certain interfering factors in clinical operations. Cerebrovascular blood flow velocity and resistance index are important reference indicators for CBF in preterm newborns (35, 38, 39). Schneitz et al. found that higher levels of ACA-RI are related to cerebral ischemia and hypoxia in premature infants (40). In this study, ACA Vd in the IVH group was significantly reduced and ACA RI was substantially increased (*P* < 0.05). We speculate that when cerebral vascular resistance increased, the CBF velocity at the end of ventricular diastole significantly decreased, and then the reduction or interruption of CBF could lead to the ischemia and hypoxia of fragile germinal matrix in preterm newborns, which ultimately led to IVH (25, 26).

For preterm infants, the larger the range of BP fluctuations, the higher the risk of developing IVH. However, it is necessary to further analyze the correlation between BPV and CBF to determine whether BPV can indirectly reflect CBF disorders caused by dysfunction of cerebral vascular autoregulation, and serve as an indicator for predicting IVH. This study investigated the correlation between abnormal BPV indicators and ACA-RI. It was found that SBPmax-min, SBP SD, SBP CV, and DBPmax-min in premature infants in the IVH group were positively correlated with ACA-RI (*p* < 0.05), while BPV indicators in non-IVH group were not correlated with ACA-RI (*p* > 0.05). We speculate that the cerebral vascular autoregulation function of preterm infants in the IVH group is impaired in the early postnatal period, presenting as pressure passive CBF, which increases the range of BP fluctuations and ACA-RI reactivity in the early stage. Hypoperfusion of CBF leads to ischemia of the germinal matrix, followed by reperfusion leading to vasodilation and bleeding (28). The cerebral vascular autoregulation function of premature infants in the non-IVH group was not affected, and they could maintain stable CBF despite BP fluctuations. Therefore, the increase in ACA-RI, which is reflected by the increase in BPV, may be an early marker of the disruption of cerebral vascular autoregulation function, which can indirectly reflect CBF fluctuations to a certain extent, consistent with the research results of Argolo et al. (41).

Preterm newborns are prone to exposure to hemodynamic interference factors and face the risk of CBF hypoperfusion and hyperperfusion injury in the early postnatal period. Multivariate logistic regression analysis found that SBP SD (OR: 1.480, 95%CI: 1.020–2.147) and ACA-RI (OR: 3.027, 95%CI 2.769–3.591) were independent risk factors for IVH in preterm newborns. This indicates that high BPV and ACA-RI status may play a critical role in the development of IVH in preterm infants, consistent with the research results of Da Costa et al. (10) and Farag et al. (35), which suggest that abnormal BP fluctuations based on optimal MABP and increased ACA-RI are closely related to IVH. ACA-RI > 0.79 with 55.1% sensitivity and 83.7% specificity, and SBP SD > 6.05 with 55.1% sensitivity and 79.1% specificity. The combined detection of SBP SD and ACA-RI had the highest AUC of 0.725, with 61.2% sensitivity and 79.1% specificity in predicting IVH. Therefore, early high BPV and ACA-RI reflect disturbances in CAR function and CBF dynamics, which have a certain reference value for early prediction of IVH, especially for severe IVH.

Our study has some limitations. First it is a single center study, with a relatively small sample size that precluded more in-depth analyses. Second, the transcranial ultrasound was not performed routinely every day, so the precise hemorrhage timing was not accurately known, and the changes in BPV and cerebral blood flow parameters before and after bleeding cannot be analyzed.

## 5. Conclusions

Our study suggests that there is a direct positive correlation between BPV and ACA-RI in IVH, and the increase in BPV and ACA-RI reflects hemodynamic disorders and abnormal CBF perfusion. SBP SD and ACA-RI are independent risk factors for the development of IVH in preterm infants. Combined detection of SBP SD and ACA-RI has a certain predictive effect on early identification of IVH. Paying close attention to BP fluctuations and CBF parameters may provide new references for the prevention and management of IVH in VLBW premature infants.

## Data Availability

The raw data supporting the conclusions of this article will be made available by the authors, without undue reservation.

## References

[1] McCrea HJ Ment LR. The diagnosis, management, and postnatal prevention of intraventricular hemorrhage in the preterm neonate. Clin Perinatol. (2008) 35(4):777–94. 10.1891/11-T-722 19026340 PMC2901530

[2] Perlman JM. Periventricular-intraventricular hemorrhage in the premature infant—a historical perspective. Semin Perinatol. (2022) 46(5):1–8. 10.1016/j.semperi.2022.151591 35422351

[3] Cizmeci MN de Vries LS Ly LG van Haastert IC Groenendaal F Kelly EN Periventricular hemorrhagic infarction in very preterm infants: characteristic sonographic findings and association with neurodevelopmental outcome at age 2 years. J Pediatr. (2020) 217:1–7. 10.1016/j.jpeds.2019.09.081 31706634

[4] Hwang-Bo S Seo YM Oh MY Im SA Youn YA. The prognosis of refractory hypotension and severe intraventricular hemorrhage in very low birth weight infants. Medicine (Baltimore). (2022) 101(30):e29598. 10.1097/MD.0000000000029598 35905281 PMC9333540

[5] Luo J L Zeng H Reis C Chen S. Research advances of germinal matrix hemorrhage: an update review. Cell Mol Neurobiol. (2019) 39(1):1–10. 10.1007/s10571-018-0630-5 30361892 PMC11469802

[6] Tran TT Veldman A Malhotra A. Does risk-based coagulation screening predict intraventricular haemorrhage in extreme premature infants? Blood Coagul Fibrinolysis. (2012) 23(6):532–6. 10.1097/MBC.0b013e3283551145 22627584

[7] Vesoulis ZA Flower AA Zanelli S Rambhia A Abubakar M Whitehead HV Blood pressure extremes and severe IVH in preterm infants. Pediatr Res. (2020) 87(1):69–73. 10.1038/s41390-019-0585-3 31578033 PMC6962547

[8] Lightburn MH Gauss CH Williams DK Kaiser JR. Observational study of cerebral hemodynamics during dopamine treatment in hypotensive ELBW infants on the first day of life. J Perinatol. (2013) 33(9):698–702. 10.1038/jp.2013.44 23619374 PMC3735635

[9] Peter DS Gandy C Hoffman SB. Hypotension and adverse outcomes in prematurity: comparing definitions. Neonatology. (2017) 111(3):228–33. 10.1159/000452616 27898415

[10] Da Costa CS Czosnyka M Smielewski P Austin T. Optimal mean arterial blood pressure in extremely preterm infants within the first 24 h of life. J Pediatr. (2018) 203:242–8. 10.1016/j.jpeds.2018.07.096 30243537

[11] Miall-Allen VM de Vries LS Whitelaw AG. Mean arterial blood pressure and neonatal cerebral lesions. Arch Dis Child. (1987) 62:1068–9. 10.1136/adc.62.10.1068 3314723 PMC1778679

[12] Vesoulis ZA El Ters NM Wallendorf M Mathur AM. Empirical estimation of the normative blood pressure in infants <28 weeks gestation using a massive data approach. J Perinatol. (2016) 369(4):291–5. 10.1038/jp.2015.185 PMC4808440 26633144

[13] Escourrou G Renesme L Zana E Rideau A Marcoux MO Lopez E How to assess hemodynamic status in very preterm newborns in the first week of life? J Perinatol. (2017) 37(9):987–93. 10.1038/jp.2017.57 28471441

[14] Zubrow AB Hulman S Kushner H Falkner B. Determinants of blood pressure in infants admitted to neonatal intensive care units: a prospective multicenter study. Philadelphia neonatal blood pressure study group. J Perinatol. (1995) 15(6):470–9. 8648456

[15] Parati G Stergiou GS Dolan E Bilo G. Blood pressure variability: clinical relevance and application. J Clin Hypertens (Greenwich). (2018) 20(7):1133–7. 10.1111/jch.13304 30003704 PMC8030809

[16] Parati G Ochoa JE Lombardi C Bilo G. Blood pressure variability: assessment, predictive value, and potential as a therapeutic target. Curr Hypertens Rep. (2015) 17(4):537–55. 10.1007/s11906-015-0537-1 25790801

[17] Papile LA Burstein J Burstein R Koffler H. Incidence and evolution of subependymal and intraventricular hemorrhage: a study of infants with birth weights less than 1,500 gm. J Pediatr. (1978) 92(4):529–34. 10.1016/s0022-3476(78)80282-0 305471

[18] Xu B Ji Q Zhang Y Shen L Cao M Cai K. Postoperative blood pressure variability exerts an influence on clinical outcome after coil embolization of ruptured intracranial aneurysms. Neurol Res. (2017) 39(9):813–8. 10.1080/01616412.2017.1348653 28675964

[19] Da J Zhang Z Shen Y Li Q Hu Y Zha Y. Blood pressure variability is independent of systolic pressure in adolescent and young adult patients undergoing hemodialysis. Pediatr Res. (2018) 83(3):615–21. 10.1038/pr.2017.293 29166378

[20] Law JB Wood TR Gogcu S Comstock BA Dighe M Perez K Intracranial hemorrhage and 2-year neurodevelopmental outcomes in infants born extremely preterm. J Pediatr. (2021) 238(10):124–34. 10.1016/j.jpeds.2021.06.071 34217769 PMC8551011

[21] Vohr BR. Neurodevelopmental outcomes of premature infants with intraventricular hemorrhage across a lifespan. Semin Perinatol. (2022) 46(5):e151594. 10.1016/j.semperi.2022.151594 35379516

[22] Garvey AA Walsh BH Inder TE. Pathogenesis and prevention of intraventricular hemorrhage. Semin Perinatol. (2022) 46(5):e151592. 10.1016/j.semperi.2022.151592 35450738

[23] Soul JS Hammer PE Tsuji M Saul JP Bassan H Limperopoulos C Fluctuating pressure-passivity is common in the cerebral circulation of sick premature infants. Pediatr Res. (2007) 61(4):467–73. 10.1203/pdr.0b013e31803237f6 17515873

[24] Rhee CJ Fraser CD Kibler K Easley RB Andropoulos DB Czosnyka M The ontogeny of cerebrovascular pressure autoregulation in premature infants. Acta Neurochir Suppl. (2014) 34(12):926–31. 10.1038/jp.2014.122 PMC4383263 25010225

[25] Rhee CJ Kaiser JR Rios DR Kibler KK Easley RB Andropoulos DB Elevated diastolic closing margin is associated with intraventricular hemorrhage in premature infants. J Pediatr. (2016) 174:52–6. 10.1016/j.jpeds.2016.03.066 27112042 PMC4925245

[26] Al-Aweel I Pursley DM Rubin LP Shah B Weisberger S Richardson DK. Variations in prevalence of hypotension, hypertension, and vasopressor use in NICUs. J Perinatol. (2010) 21(5):272–8. 10.1038/sj.jp.7210563 11536018

[27] Versmold H Kitterman J Phibbs R Gregory GA Tooley WH. Aortic blood pressure during the first 12 h of life in infants with birth weight 610 to 4,200 grams. Pediatrics. (1981) 67(5):607–13. 10.1542/peds.67.5.607 7254989

[28] Bada HS Korones SB Perry EH Arheart KL Ray JD Pourcyrous M Mean arterial blood pressure changes in premature infants and those at risk for intraventricular hemorrhage. J Pediatr. (1990) 117(4):607–14. 10.1016/s0022-3476(05)80700-0 2213390

[29] Lou HC Skov H Pedersen H. Low cerebral blood flow: a risk factor in the neonate. J Pediatr. (1979) 95(4):606–9. 10.1016/S0022-3476(79)80779-9 480043

[30] Lampe R Rieger-Fackeldey E Sidorenko I Turova V Botkin N Eckardt L Assessing key clinical parameters before and after intraventricular hemorrhage in very preterm infants. Eur J Pediatr. (2020) 179(6):1–9. 10.1007/s00431-020-03585-9 31993776 PMC7220978

[31] Bel FV Bor MVD Stijnen T Baan J Ruys JH. Aetiological rôle of cerebral blood-flow alterations in development and extension of peri-intraventricular haemorrhage. Dev Med Child Neurol. (1987) 29(5):601–14. 10.1111/j.1469-8749.1987.tb08502.x 3311857

[32] Huang X Guo H Yuan L Cai Q Zhang M Zhang Y Blood pressure variability and outcomes after mechanical thrombectomy based on the recanalization and collateral status. Ther Adv Neurol Disord. (2021) 14:1–13. 10.1177/1756286421997383 PMC7940733 33747130

[33] Da Costa CS Czosnyka M Smielewski P Mitra S Stevenson GN Austin T. Monitoring of cerebrovascular reactivity for determination of optimal blood pressure in preterm infants. J Pediatr. (2015) 167(1):86–91. 10.1016/j.jpeds.2015.03.041 25891381

[34] Menke J Michel E Rabe H Bresser BW Grohs B Schmitt RM Simultaneous influence of blood pressure, PCO2, and PO2 on cerebral blood flow velocity in preterm infants of less than 33 weeks’ gestation. Pediatr Res. (1993) 34(2):173–7. 10.1203/00006450-199308000-00014 8233721

[35] Farag MM Gouda MH Almohsen AMA Khalifa MA. Intraventricular hemorrhage prediction in premature neonates in the era of hemodynamics monitoring: a prospective cohort study. Eur J Pediatr. (2022) 181(12):4067–77. 10.1007/s00431-022-04630-5 36171508 PMC9649466

[36] Azhibekov T Noori S Soleymani S Seri I. Transitional cardiovascular physiology and comprehensive hemodynamic monitoring in the neonate: relevance to research and clinical care. Semin Fetal Neonatal Med. (2014) 19(1):45–53. 10.1016/j.siny.2013.09.009 24555196

[37] Pereira SS Sinha AK Shah DK Kempley ST. Common carotid artery blood flow volume in extremely preterm infants. Acta Paediatr. (2021) 110(4):1157–65. 10.1111/apa.15655 33145798

[38] Altit G Bhombal S Chock VY. Cerebral saturation reflects anterior cerebral artery flow parameters by Doppler ultrasound in the extremely premature newborn. J Perinatol. (2022) 42(2):1–6. 10.1038/s41372-021-01145-z 34247188

[39] Pazandak C Mir IN Brown LS Chalak LF. Placental pathology, cerebral blood flow, and intraventricular hemorrhage in preterm infants: is there a link? Pediatr Neurol. (2020) 108:65–9. 10.1016/j.pediatrneurol.2020.01.001 32451157

[40] Baik-Schneditz N Höller N Urlesberger B Schwaberger B Schmölzer GM Pichler G. Cerebral Doppler resistance Index (RI) is associated with regional cerebral oxygenation. Acta Paediatr. (2020) 109(11):2299–301. 10.1111/apa.15318 32304596 PMC7687206

[41] Argollo N Lessa I Ribeiro S. Cranial Doppler resistance index measurement in preterm newborns with cerebral white matter lesion. J Pediatr. (2006) 82(3):221–6. 10.2223/JPED.1488 16773177

